# Grateful Thanks to Our War Correspondents

**Published:** 1914-08-22

**Authors:** 


### HOSPITALS AND THE WAR.

### Grateful Thanks to our War Correspondents.

**We are genuinely grateful for the response which has been made to our request addressed to the hospital managers and officials throughout the country. A number of gentlemen have promptly accepted our invitation to become "The Hospital's" correspondents in the centre of population in which their work and hospital are situated. Many of them have been kind enough to send us newspaper cuttings and other documents and papers which bear on their work, and nearly the whole of the information which we are enabled to publish in the pages which follow has been contributed as original matter by our correspondents. We are sure that our readers, and all who take a genuine interest in the steps which are being taken to secure adequate provision and treatment for the sick and wounded brought to these shores, will share our gratitude for the generous and prompt action of the gentlemen in question. We hope to arrange to have a correspondent in every important county division throughout England and Scotland, and we propose in our next issue, when we have had time to prepare a careful list of all who have consented to become correspondents, to be in a position to state where and in what places vacancies still exist. We hope that all our correspondents will accept this grateful recognition on the part of the Editor of the valuable services they are rendering, and that they will understand that full credit will be given to them for all the contributions they send, and that suitable acknowledgment will be made in each case in due course.**

